# The Oxygen Reduction Reaction in Ca^2+^‐Containing DMSO: Reaction Mechanism, Electrode Surface Characterization, and Redox Mediation**

**DOI:** 10.1002/cssc.202001605

**Published:** 2020-09-18

**Authors:** Pawel Peter Bawol, Philip Heinrich Reinsberg, Andreas Koellisch‐Mirbach, Christoph Johannes Bondue, Helmut Baltruschat

**Affiliations:** ^1^ Institut für Physikalische und Theoretische Chemie Universität Bonn Römerstraße 164 53117 Bonn Germany

**Keywords:** Ca−O_2_ battery, differential electrochemical mass spectrometry, disproportionation, oxygen reduction reaction, X-ray photoelectron spectroscopy

## Abstract

In this study the fundamental understanding of the underlying reactions of a possible Ca−O_2_ battery using a DMSO‐based electrolyte was strengthened. Employing the rotating ring disc electrode, a transition from a mixed process of O_2_^−^ and O_2_^2−^ formation to an exclusive O_2_^−^ formation at gold electrodes is observed. It is shown that in this system Ca‐superoxide and Ca‐peroxide are formed as soluble species. However, there is a strongly adsorbed layer of products of the oxygen reduction reaction (ORR) s on the electrode surface, which is blocking the electrode. Surprisingly the blockade is only a partial blockade for the formation of peroxide while the formation of superoxide is maintained. During an anodic sweep, the ORR product layer is stripped from the electrode surface. With X‐ray photoelectron spectroscopy (XPS) the deposited ORR products were shown to be Ca(O_2_)_2_, CaO_2_, and CaO as well as side‐reaction products such as CO_3_^2−^ and other oxygen‐containing carbon species. It is shown that the strongly attached layer on the electrocatalyst, that was partially blocking the electrode, could be adsorbed CaO. The disproportionation reaction of O_2_^−^ in presence of Ca^2+^ was demonstrated via mass spectrometry. Finally, the ORR mediated by 2,5‐di‐*tert*‐1,4‐benzoquinone (DBBQ) was investigated by differential electrochemical mass spectrometry (DEMS) and XPS. Similar products as without DBBQ are deposited on the electrode surface. The analysis of the DEMS experiments shows that DBBQ^−^ reduces O_2_ to O_2_^−^ and O_2_^2−^, whereas in the presence of DBBQ^2−^ O_2_^2−^ is formed. The mechanism of the ORR with and without DBBQ is discussed.

## Graphical abstract

**Ca−O_2_ redox chemistry**: For the development of metal‐air batteries an understanding of the reaction mechanism is necessary. This study characterizes the electrochemical processes during oxygen reduction and evolution reactions (ORR and OER) in Ca^2+^‐containing DMSO and formulates a mechanistic model for the ORR. Additionally, the functionality of 2,5‐di‐*tert*‐1,4‐benzoquinone (DBBQ) as a redox mediator for ORR is presented.

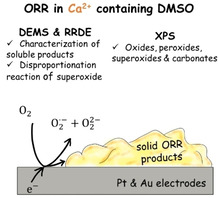

## Introduction

To overcome future energy storage problems, several different technologies will be needed, among which batteries will potentially be a key player for mobile applications and transportation. Considering the scarcity of several elements used in today's lithium ion batteries (e. g., cobalt)^[1]^ and, even more importantly, socio‐economic impacts of, for example, cobalt mining,^[2]^ alternative battery technologies have to be developed to unleash the full potential of electrochemical energy storage. One possibility is to use other chemistries such as metal‐air and metal‐sulfur, which do not necessarily require the use of cobalt catalysts. Another problem for lithium technology is the lack of the resource lithium and the water‐consuming mining.^[3]^ On the other hand, calcium, being the fifth‐most abundant metal on earth, combines a high abundance with a competitive volumetric capacity of 2072 mAh· cm^−3[4,5]^ and thus is a promising candidate as anode material in future battery applications. Early studies, however, showed the difficulties of Ca plating/stripping.^[6]^ Fortunately, more recent studies revealed electrochemical systems in which the Ca plating/stripping becomes accessible.[^4^, ^5^, ^7^, ^8^]

**Figure 1:**
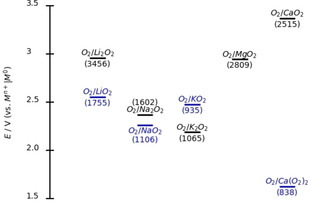
Thermodynamics of alkali superoxides and peroxides in comparison to calcium superoxide and calcium peroxide (all in solid state). The standard potentials are given with respect to the corresponding metal of the superoxide/peroxide. Based on these potentials the theoretical specific energy (in Wh kg^−1^) with respect to the mass of the product is displayed in parentheses. To our knowledge no thermodynamic data is available for the O_2_/Mg(O_2_)_2_ system. Note the O_2_/Ca(O_2_)_2_ system as it was previously miscalculated.^[13]^ The correct value is displayed here.

Combining Ca plating/stripping as anode reaction with an oxygen cathode promises impressive theoretical specific energies . This kind of battery, in which a metal anode is combined with an oxygen cathode, was extensively investigated, and several combinations of alkali metals and oxygen have been proposed.[^9^, ^10^, ^11^] It is interesting that even on good catalysts for the oxygen reduction reaction (ORR) in aqueous media, such as platinum, where oxygen is reduced to water and thus the dioxygen bond is broken, the situation is completely changing if a non‐aqueous electrolyte is used.^[12]^ There, the reaction typically stops at the superoxide or peroxide stage. Figure 1 shows the theoretical open‐circuit voltage (OCV) of the formation of lithium, sodium, and potassium superoxides and peroxides in comparison to calcium superoxide and calcium peroxide. The resulting theoretical specific energy in Wh kg^−1^ is also displayed in Figure 1 (see numbers in parentheses).

Figure 1 shows that especially the O_2_/CaO_2_ system delivers the second‐highest theoretical specific energy density for the displayed systems. O_2_/Ca(O_2_)_2_ shows the lowest theoretical specific energy with 838 Wh kg^−1^. But even this value is higher than the value for the Li‐ion technology (C/LiCoO_2_) with a theoretical specific energy density of 387 Wh kg^−1^.^[14]^ Preceding studies on the ORR and oxygen evolution reaction (OER) in the Ca^2+^ system in DMSO were already done by us.[^13^, ^15^] There, we observed a significant effect of the electrocatalyst on the ORR mechanism. On Au electrodes the formation of peroxide was observed via differential electrochemical mass spectrometry (DEMS). In contrast to that, superoxide is the main product on Rh, Pt, Ru, and glassy carbon. Further investigations of the system on Pt and glassy carbon electrodes were done using DEMS in a generator–collector arrangement and rotating ring disc electrodes (RRDE). There, we found that roughly 90 % of the ORR product is soluble O_2_^−^. Taking also the amount of insoluble products into account, we observed a remarkable reversibility for a metal‐air system of 95 %. In addition, cyclic voltammetry (CV) studies unraveled that O_2_^−^ forms a contact ion pair with Ca^2+^, which was also found in several other metal−O_2_ systems.[^16^, ^17^] In a future study, we will present more evidence on this.^[18]^

In the current paper, we used the RRDE technique to get more insights into the reaction mechanism of the ORR in Ca^2+^‐containing DMSO. The homogenous disproportionation of O_2_^−^ in the presence of Ca^2+^ was investigated using MS. For the noble metals Au and Pt, ex situ X‐ray photoelectron spectroscopy (XPS) combined with Ar^+^ etching was performed to analyze the deposited ORR products on the electrode surface. Finally, we used the well‐known redox mediator in Li−O_2_ systems, 2,5‐di‐*tert*‐1,4‐benzoquinone (DBBQ), to investigate the applicability in the Ca^2+^ system by using DEMS and XPS.

## Experimental Section

### Chemicals

Calcium perchlorate tetrahydrate (99 %, Sigma Aldrich) was dried under reduced pressure at 356 K in a Büchi‐oven for 48 h. Extra‐dry DMSO (99.7 %, over molecular sieve, Acros Organics) and potassium superoxide (Acros Organics) were used as received. As supporting salt for the reference electrolyte AgNO_3_ (>99.5 %, ChemPure) was used. All electrolyte preparations were made in an Ar‐filled (Air Liquid, 99.999 %) glovebox by GS.

### Electrochemical treatment of the noble metal electrodes

Prior to the measurements in the organic solvents the noble metal electrodes (Au and Pt) were checked for cleanness. This was done by cycling the electrode in 0.5 m H_2_SO_4_ until the typical hydrogen adsorption/desorption region (for Pt) and the oxide formation (for Pt and Au) was observed in CV. Afterwards the crystals were washed with MilliQ water (18.2 MΩ) and dried under reduced pressure until further electrochemical measurements were performed.

### RRDE experiments

The RRDE measurements were performed in a closed H‐cell. The H‐cell was purged with an Ar/O_2_ mixture throughout the experiment to saturate with oxygen and avoid contamination of the electrolyte with water from the ambient air. A silver wire in a solution of 0.1 m AgNO_3_ in DMSO was used as reference electrode. To avoid contamination of the working electrolyte with AgNO_3_ the contact between reference electrode and working compartment was established via the wet surface of a closed glass stopcock. The water content of the electrolyte determined via Karl‐Fischer titration was typically 40 ppm. A gold‐disk platinum‐ring electrode with a geometric surface area of 0.196 cm^2^ (disk area) and a collection efficiency of 0.25 was used throughout the investigation.

### DEMS experiments

DEMS experiments were performed with a home‐built differentially pumped MS as described by Wolter and Heitbaum^[19]^ and Baltruschat.^[20]^ The spectrometer was connected via a flexible vacuum steel tube to a MBraun glovebox filled with a 20 : 80 O_2_/Ar atmosphere. The water content in this glovebox never exceeded a value of 0.3 ppm during the experiments. A thin‐layer DEMS cell, which was optimized for the use in metal−O_2_ systems, was used for the electrochemical experiments. In this cell, we used a porous Teflon membrane with sputter‐deposited Au as working electrode, which was interfacing the vacuum of the MS. Because of the hydrophobicity and small pore sizes of 20 nm, liquids do not penetrate through the membrane. A photograph of the electrode is shown in the Supporting Information. The wall opposite to the working electrode of the thin‐layer cell was formed by a porous polytetrafluoroethylene (PTFE) membrane interfacing an oxygen atmosphere, thus allowing continuous oxygen flow to the working electrode. Three counter electrodes (Au wires) and a reference electrode were connected via capillaries to the working electrode compartment. As reference electrode a silver wire immersed into 0.1 m AgNO_3_ in DMSO was used. The DEMS cell was operated without convection so that reaction products which are soluble in the electrolyte could accumulate in the working electrode compartment (*V*=5.6 μL). For a more detailed description of the experimental setup, see Ref. [21]. The calibration constant for oxygen was experimentally determined by performing the ORR in tertbutylammonium perchlorate‐containing DMSO. There, oxygen is exclusively reduced to superoxide.[^22^, ^23^] Thus, the calibration constant can be calculated from the ratio of the ionic current detected in the MS to the Faraday current at the electrode. Using this calibration constant, the number of electrons transferred per oxygen molecule can be evaluated. This quantity is often described in the continuous text as “one‐electron process” (*z*=1) or “two‐electron process” (*z*=2). This term refers exclusively to the above‐mentioned value and does not imply any statement about the kinetics of the electron transfer. It rather describes the stoichiometry of the reaction.

### Detection of the homogenous disproportionation of superoxide via MS

A vessel containing 25 mL of DMSO with 0.1 g KO_2_ was prepared in an argon‐filled glovebox. The vessel was closed with a rubber septum and transferred to the MS. There, the solution was continuously stirred during the experiment with a magnetic stirrer. Two cannulas were pierced through the septum. Via one, highly pure Ar gas was flushed through the experimental setup; the other one connected the gas phase of the vessel to the differentially pumped MS. Via a leak valve, the pressure in the vacuum was adjusted to 7· 10^−5^ mbar. 1 m Ca(ClO_4_)_2_ in DMSO and 1 m LiClO_4_ in DMSO were prepared in the glovebox. 3 mL of the respective solution was transferred in a sealed syringe to the experimental setup. There, the solution was right away inserted into the vessel via the septum. A sketch of the experimental setup is shown in the Supporting Information.

### XPS analysis

To investigate the chemical state of sample surfaces, XPS was used. In general, the samples were Pt or Au electrodes (*d*=10 mm), which were modified in an electrochemical experiment. The sample electrodes were mounted on a crystal holder manufactured out of steel. After the electrochemical experiment, the samples were washed with dry DMSO (99.7 %, over molecular sieve, Acros Organics) and mounted into a homemade sample‐transfer system. This transfer system allowed the transfer of a sample between the glovebox and the ultra‐high vacuum (UHV) chamber without contact to air. The XPS was part of a homemade UHV chamber with a base pressure of 5· 10^−10^ mbar.[^24^, ^25^, ^26^] The X‐ray source was a non‐monochromatized Mg K_α_ (1253.6 eV) source. As electron‐energy analyzer a hemispherical electron analyzer (Omnicron NanoTechnology EA 125) was used. Survey spectra were recorded with a pass energy of 50 eV and an energy resolution of 0.5 eV. High‐resolution spectra were recorded with a pass energy of 15 eV and an energy resolution of 0.1 eV. To increase the signal‐to‐noise ratio, the high‐resolution spectra are an average of nine spectra. By doing this, the resolution of our device was determined to be 1.07 eV [measured with the full width at half maximum (FWHM) of the Au 4f_7/2_ peak]. The binding energy was calibrated using the Au 4f_7/2_ peak at 83.95 eV or the Pt 4f_7/2_ peak at 71.09 eV,^[27]^ which were present in all recorded spectra. The XPS measurements were accompanied by Ar^+^ etching (Physical Electronics Model 04–191, 3 kV, *I*_emission_=25 mA, *I*_sample_=1 μA) The electrochemical experiments were performed in a glovebox filled with a 80 : 20 Ar/O_2_ mixture. The humidity in the glovebox never exceeded a value of 0.3 ppm. Our experiments showed that the deposited films of electrically non‐conducting species were thin and in good contact with the conducting Au or Pt crystal. Therefore, no charge compensation with an electron flood gun was needed.

## Results and Discussion

### RRDE and DEMS investigations of the ORR in Ca(ClO_4_)_2_ in DMSO

**Figure 2:**
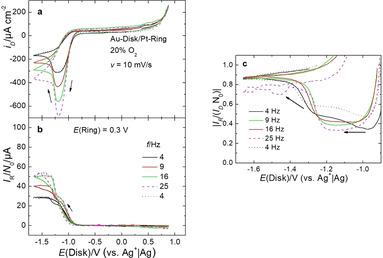
ORR in presence of Ca^2+^ at a gold‐RRDE. a) Currents at the gold‐disk. b) Corresponding currents at a platinum‐ring. c) Collection efficiency. Electrolyte: 0.1 m Ca(ClO_4_)_2_ in DMSO. *A*(Disk)=0.196cm^2^, *N*_0_=0.25.

The ORR at a gold electrode in 0.1 m Ca(ClO_4_)_2_ is shown in Figure 2. After a rise in current at a potential of −0.8 V, a plateau is observed for a rotation rate of 4 Hz, which is close to the diffusion‐limited current for a two‐electron reduction of oxygen (based on the Levich equation) and thus agrees well with the previous observations made in the DEMS flow‐through cell.^[15]^

However, after a charge flow of 8200 μC cm^−2^, the current starts to become less negative at a potential of −1.29 V and reaches a second plateau, which agrees well with the diffusion‐limited current of the one‐electron process. In this potential range we observe in a plot of the collection efficiency as a function of the disk potential (Figure 2c) a transition to a value close to 1, and thus, a preferred formation of soluble species. The apparent collection efficiency is herein defined as the ratio of the ring current $I_{R}$ to the disc current $I_{D}$ normalized to the theoretical collection efficiency of the disk elektode $N_{0}$ ($\frac{I_{R}}{I_{D}\;N_{0}\;}$ ). This is in principle reminiscent of the ORR in Li^+^‐containing DMSO, where a transition to superoxide formation was observed after the electrode was partially blocked by Li_2_O_2_.[^28^, ^29^] However, a major difference between these measurements is the large charge which can be passed before this transition occurs: Even if the charge detected at the ring electrode is subtracted (2500 μC cm^−2^), the remaining charge is still 5700 μC cm^−2^ and thus in the order of several monolayers. Since it is well known that insoluble peroxides and superoxides are insulating and thus poison the electrode surface, this result implies that in fact most of the reduction charge is passed into soluble species. Considering that the charge detected at the ring only accounts for a small portion of the produced species, this can only be understood by assuming that the soluble species are not readily oxidizable at the ring.^[30]^ Here, we want to point out that the previously reported CVs in the DEMS cells showed a plateau for the 2 e^−^/O_2_ process in the ORR.^[13]^ This difference to the measurement shown here is due to the higher convection in the RRDE experiment and thus a higher flowing charge, which is sufficient to poison the electrode and trigger the transition from the 2 e^−^/O_2_ process to the 1 e^−^/O_2_ process.

**Figure 3:**
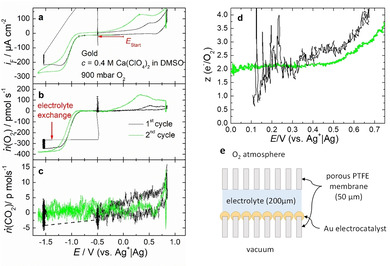
Thin‐layer DEMS measurement of ORR on porous Au/PTFE in presence of Ca^2+^. a) Currents at the gold working electrode. b) Corresponding flux of oxygen. c) Corresponding flux of CO_2_. d) Number of electrons transferred per evolved molecule of O_2_. Black: After holding the potential for 500 s at −1.5 V, the electrolyte was exchange under potential control and then stepped to −0.5 V before continuing cycling. Green: DEMS measurement without potential step and without electrolyte exchange. Electrolyte: 0.4 m Ca(ClO_4_)_2_ in DMSO, 900 mbar O_2_. The diffusion‐limited currents for oxygen consumption in the absence of convection are due to the special thin‐layer construction of the cell. e) Cross section of the components that form the electrolyte volume of the thin layer cell. A photograph of the electrode is shown in the Supporting Information. For more details about the DEMS electrochemical cell, see Ref. [21].

Using the DEMS thin‐layer cell in stagnant electrolyte, it can also be shown that the peroxide formed during the ORR is soluble in the DMSO‐based electrolyte by examining the electron number of the OER (see Figure 3, the oxidation of a peroxide corresponds to a two‐electron process). To probe for soluble, reduced oxygen species, the experiment was carried out as follows: First, the potential was swept to −1.5 V, where it was kept for 500 s, and roughly 170 nmol of O_2_ was reduced. Then, the electrolyte was exchanged, and the potential was stepped to −0.5 V before it was cycled to 0.75 V. The amount of O_2_ evolved during the anodic sweep was only 1.5 nmol. In contrast to this, the amount of O_2_ reduced in an experiment without a potential stop and without electrolyte exchange was 42 nmol, and the amount evolved was 12 nmol (the difference between ORR and OER charge is probably caused by the transport of the soluble species into the capillaries of the cell). The large discrepancy between OER and ORR charge in the case of electrolyte exchange implies that the peroxide species (as indicated by the two‐electron process during reduction as well as oxidation) are at least partially soluble.

Since we have shown that the ORR products are soluble, it has to be shown if it is possible to reactivate the electrode by dissolving the ORR products. The reactivation of the electrode was investigated with the RRDE. The convection induced by the RRDE should favor the dissolution of the ORR products.

**Figure 4:**
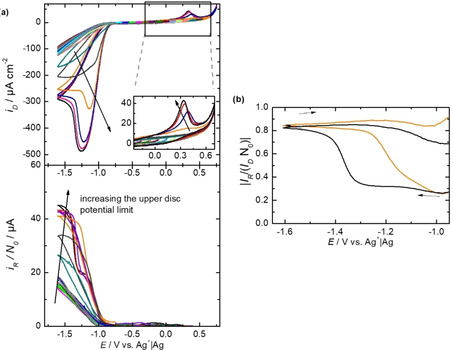
a) RRDE study with a partially blocked Au electrode at 20 mV s^−1^. The upper potential limit is increased by 100 mV per cycle. The arrows indicate the reactivation of the electrode due to a potential opening. In all measurements 0.1 m Ca(ClO_4_)_2_ in DMSO with 20 % O_2_ was used. The rotation frequency is in all measurements was 9 Hz. The roughness factor of the disk electrode was 3. b) Calculated apparent transfer efficiency for the black‐ and orange‐traced measurement in (a) in the potential range of the ORR.

The electrode was first blocked by a potential stop in the ORR. Then a CV was recorded in the potential range of the ORR (see Figure 4a). Even with a blocked electrode we observe a faradaic current for the ORR. An explanation for this might be the electro‐migration of ions through the blocking insulating layer if higher field strengths are applied as we previously reported for the ORR in Mg^2+^‐containing DMSO.^[31]^ The potential window is successively opened positively by 100 mV. Reaching a potential of 0.3 V for the upper limit, the reactivation of the electrode can be recognized by an increase in the reduction current of the ORR (see arrows in Figure 4). At the same time, an oxidation peak is obtained at 0.3 V, which can be attributed to the oxidation of products deposited on the electrode (see magnification of the OER region in Figure 4). A further opening of the potential regenerates the electrode completely and shows the necessity of applying higher potentials for the reactivation of the electrode. This measurement shows that the electrode is not simply regenerated by dissolution of the ORR products, but that a blocking surface layer (possibly an adsorbate) remains on the surface, which can only be stripped at potentials around 0.3 V. In the first cycle reaching this potential, this stripping is rather incomplete, as can be seen in the subsequent sweep into the ORR region. The more the blocking is lifted, the larger grows the corresponding oxidation peak and also shifts to lower potentials. This is indicative of a nucleation and growth behavior: Oxidation of this layer is slow and only occurs at defects on the boundary between the layer and the free surface sites.

As the disc electrode is reactivated the amount of detected superoxide at the ring electrode increases (see Figure 4a). For the completely reactivated electrode (see black traced measurement in Figure 4a), again a transition from the formation of insoluble species to the formation of soluble species is observed, as can be seen in the apparent collection efficiency in Figure 4b. For a partially blocked electrode (see orange‐traced measurement in Figure 4a) the formation of superoxide shifts 200 mV in positive direction (see orange‐traced apparent collection efficiency in Figure 4b). This shows that the peroxide formation preferentially occurs at active sites on the electrode, which are already blocked in the orange‐traced measurement, and thus the superoxide formation starts earlier during the sweep.

### Disproportionation of superoxide in the presence of Ca^2+^

**Figure 5:**
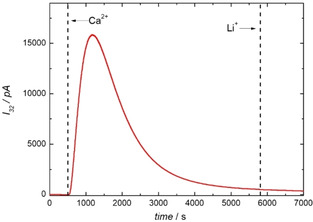
Ionic current of mass 32 (red) as a function of time. The gas phase over a stirred solution containing 0.1 g KO_2_ in 25 mL DMSO was analyzed by MS after adding 3 mL of 1 m Ca(ClO_4_)_2_ and 1 m LiClO_4_ in DMSO. The points in time at which the Ca^2+^‐ and Li^+^‐containing DMSO were added, are indicated as dashed line in the graphs. For details on the experimental setup see Figure S1 in the Supporting Information.

Another known reaction in non‐aqueous metal‐air batteries is the disproportionation of the superoxide. The common procedure to test if superoxide undergoes a disproportionation in the presence of a cation of interest is to use a solution of the stable superoxide compound KO_2_ (in this study: KO_2_ in DMSO) and add a solution containing the cation of interest [in this study: Ca(ClO_4_)_2_ in DMSO].[^32^, ^33^, ^34^] The products of the disproportionation of O_2_^−^ are O_2_^2−^ and O_2_. Hence, this reaction can be followed by measuring the ionic current of mass 32 as shown in Figure 5.

After adding 3 mL of 1 m Ca(ClO_4_)_2_ in DMSO to 0.1 g KO_2_ in 25 mL DMSO we observe an increase of the ionic current of mass 32. Further on the ionic current of mass 32 goes through a maximum and fades within 2 h. This shows that O_2_^−^ undergoes disproportionation in the presence of Ca^2+^. Adding again 3 mL of 1 m LiClO_4_ in DMSO to the solution does not increase the ionic current of mass 32 further, which shows that the disproportionation of O_2_^−^ is finished. Otherwise, addition of Li^+^ should lead to another increase in the signal on mass 32 as the remaining superoxide will disproportionate under the formation of O_2_ and insoluble Li_2_O_2_.

Astonishingly, parallel to oxygen formation, ion current transients corresponding to volatile species like H_2_O (*m*/*z*: 18), H_2_CO (*m*/*z*: 30), CO (*m*/*z*: 28), CO_2_ (*m*/*z*: 44), and SO_2_ (*m*/*z*: 64) were observed (see Figure S2 in the Supporting Information). To our knowledge this was not reported so far in this kind of experiment or in DEMS experiments in metal‐air systems during the ORR. A reason why we observed these signals might be the sensitivity of our experiment, which was optimized by using the differentially pumped vacuum system as well as a relatively high pressure in the vacuum system that was adjusted by the leak valve. The formulation of a mechanism for the formation of these compounds during disproportionation is currently not possible. The most plausible source would be a side reaction with singlet oxygen, which was observed as a by‐product of the disproportionation reaction in presence of various cations in significant amounts.[^34^, ^35^] Due to the high reactivity of singlet oxygen, it is plausible that DMSO is decomposed in a follow‐up reaction under generation of the above species. Another side product of the disproportionation that was already reported is CO_3_^2−^.[^34^, ^36^] We proved the presence of carbonates after the disproportionation by acidifying the solution with H_2_SO_4_ and observed a CO_2_ formation from the chemical reaction, as was also done by Mahne et al. (see Figure S3 in the Supporting Information).^[35]^

### XPS studies of the electrode surfaces

The Pt and Au electrodes after the ORR were characterized by XPS at various stages of Ar^+^‐etching to get insights into the depth‐profile of elemental distribution of the deposited film. The electrochemical experiments prior to the XPS measurements are described in the Supporting Information.

**Figure 6:**
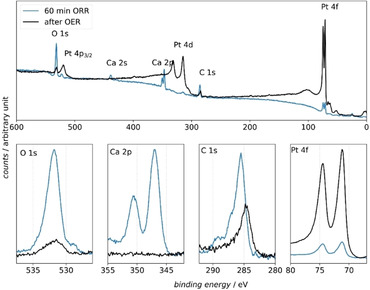
Top figure: Survey spectra of a Pt electrode after performing the ORR at −1.5 V vs. Ag^+^|Ag for 60 min (blue line) and after sweeping the potential into the OER (black line). Bottom figures: High‐resolution XP spectra of the O 1s, Ca 2p, C 1s, and Pt 4f region. The associated electrochemical experiment is shown in Figure S4 in the Supporting Information.

First of all the survey spectra of the Pt electrode after the ORR and after the OER are displayed in Figure 6.

After holding the electrode potential in the ORR region, the spectrum for the Pt electrode surface mainly shows peaks at binding energies that contribute to the core levels of carbon, calcium, and oxygen. The Pt peaks are also visible in the measurement after the ORR, and especially the 4f peaks of Pt show an interesting feature: The increase of the intensity towards higher binding energies after the 4f peaks is an indication of inelastic scattering of the Pt 4f electrons. This shows that Pt is buried under a layer of precipitated products of the ORR.^[37]^ Also, in the region in which the Pt 4d peaks are expected to appear (between 315 and 332 eV binding energy) the baseline increases. This again shows that the X‐rays excite the Pt 4d core levels and that the emitted photoelectrons are inelastically scattered as they are passing through the deposited thin layer. We made the same observation using an Au electrode (see Figure S5 in the Supporting Information). The surface sensitivity of the XPS experiment and the fact that the 4f peaks of the electrode material are observed, give evidence that a thin film is deposited on the electrode surface. After a sweep into the OER region, the Pt peaks are largely increased, indicating that the surface now is only covered by a very thin film. This will be discussed below.

**Figure 7:**
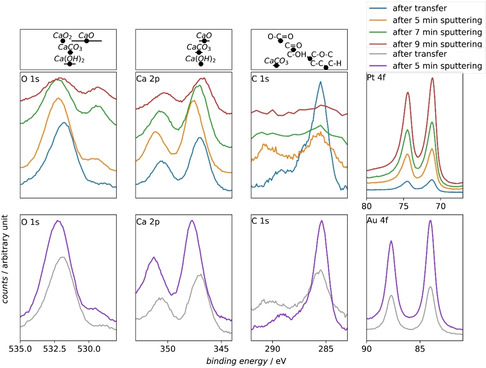
High resolution XP spectra of various binding energy regions. The corresponding core shell orbitals are indicated in the plots. The XP spectra are recorded after 60 min of ORR in 0.2 m Ca(ClO_4_)_2_ in DMSO on a Pt electrode (middle row) and an Au electrode (bottom row). The different spectra are recoded after different Ar^+^ etching times as indicated in the legend. Typical binding energy values for different chemical compounds are displayed in the first row. The displayed values are the average values (points) with the standard deviation from the average. (error bar). An overview of the different values from the different references is shown in the Supporting Information.

High‐resolution XP spectra of the O 1s, Ca 2p, C 1s, and 4f peaks of the electrode material (Au and Pt) were recorded and are shown for different Ar^+^ etching times in Figure 7. In addition, a literature survey on binding energies of CaO, Ca(OH)_2_, CaCO_3_, and CaO_2_ was done. The results are plotted in the first row of Figure 7. The solid dots in Figure 7 indicate the average of the binding energies available in literature, while the error bar denotes the standard deviation. The values used and references are summed up in the Supporting Information. Figure 7 also shows common binding energies of different carbon species.

**Figure 8:**
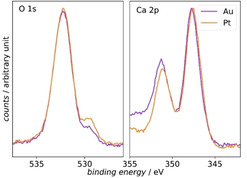
Comparison of the XP spectra in the O 1s region and Ca 2p region of the experiments on the Au and Pt electrode. The spectra are both recorded after 5 min Ar^+^ etching. For the Ca 2p region the counts were normalized to the Ca 2p_3/2_ peak. The O 1s region is displayed with no further normalization procedure.

Comparing the XP spectra in Figure 7 for the Pt and the Au electrode after the transfer and after 5 min Ar^+^ treatment shows that the O 1s and Ca 2p peaks are observed at similar binding energies on the different electrode materials. This is an indication that the same species are deposited on both electrode materials. Only in the C 1s region in the spectra collected on the Pt electrode a shoulder is visible at a binding energy of 287.3 eV, indicating that more C−O−C and C−OH species are present on the surface. The comparable chemical state regarding the calcium oxygen compounds of the film deposited during ORR becomes clearer in Figure 8, where the O 1s and Ca 2p XP spectra after 5 min Ar^+^ etching for the Au and the Pt electrode are plotted. The O 1s regions of the peak at a binding energy of 532.3 eV overlap. This shows that the same amount of oxygen is present in both experiments. The rather large FWHM of 2.34 eV of the peak at 523.3 eV indicates that this peak probably contains excitations from the O 1s core level out of different chemical environments and thus, different chemical compounds. In both spectra, the additional small peak at a binding energy of 529.5 eV shows the presence of another oxygen species with a higher electron density on both electrode materials. The peaks in the Ca 2p region also appear at the same binding energies, again indicating that the same calcium oxygen species are deposited on the Au and Pt electrode. The higher intensity of the Ca 2p region towards higher binding energies is due to the superposition of the Au 4d peak and the Ca 2p peak (see survey spectra in Figure S4).

Since we have shown that the chemical composition of the ORR products on the gold and platinum electrode are the same, the further detailed analysis of the XP data will deal with the Pt electrode. The elemental composition of the film on the Pt electrode was calculated using atomic sensitivity factors and assuming a uniform distribution of the elements in the investigated volume of photoelectron formation.^[38]^ The calculated values are summed up in Table 1. Table 1 shows that carbonaceous species are located on the surface of the deposited film. After a total etching time of 7 min the C 1s signal vanishes (C 1s atomic ratio is less than 5 % of the total film, estimated from the survey spectra in Figure S6), thus inside the film the amount of carbonaceous species can be neglected. The film gets thinner through the Ar^+^ etching, as indicated by the increase of the Au 4f and Pt 4f peaks. Close to the electrode surface, the film consists only of calcium and oxygen.

**Table 1:** Surface composition (atomic ratio) calculated from the signal area of the high‐resultion spectra of the experiments with the Pt electrode shown in Figure 7. This was done by assuming a uniform elemental distribution of the volume from which photoelectrons are emitted.

*t*(Ar^+^) [min]	C[%]	Ca[%]	O [%]
0	40.2	15.3	44.4
5	20.3	23.9	55.8
7	0.0^[a]^	29.8	70.2
9	0.0^[a]^	33.3	66.7

[a] Due to the lower sensitivity of the experimental setup while recoding high‐resolution spectra it was not possible to detect carbon, and therefore this value was set to zero. The survey spectra show that by doing this the absolute error is less than 5 %.

Moreover, the Ca 2p, O 1s, and C 1s XP spectra are showing a shift in the binding energy for the different Ar^+^ etching times. This implies a change in the oxidation state of the deposited film. A detailed discussion of the O 1s and C 1s region is made by considering the deconvolution of the recorded spectra. To our knowledge, there are no reports about binding energies of the Ca 2p core level of CaO_2_ and Ca(O_2_)_2_ in literature. However, binding energies for the O 1s levels for the peroxide are available. These two compounds are expected to be the main ORR products,[^13^, ^15^] but the minor formation of CaO is also possible. In addition, the shift in binding energies of the Ca 2p core level is not as big as the shift of binding energies of the O 1s and C 1s core level, which from a chemical point of view is reasonable since in all calcium−Oxygen compounds Ca has the formal oxidation state +II. Therefore, a further discussion of the binding energy shift of the Ca 2p core level is not made here, but we will use the overall intensity of this excitation for quantification below. The C 1s region was also investigated in detail (see Figure S8 in the Supporting Information). There we observed contributions of C−O, O−C=O, and CO_3_^2−^ species. These species are located on the surface of the deposited film and disappear after Ar^+^ treatment. From previous studies on metal‐air batteries the formation of decomposition products like CO_3_^2−^ is well known, and thus it is not surprising to also find these species in the present system.[^39^, ^40^, ^41^, ^42^] As source of the carbonate we would refer to the disproportionation of superoxide and the associated side reactions (presumably through the formation of singlet oxygen), as we also discussed in the context of Figure 5.

In the survey spectrum we observe a peak at 161.5 eV binding energy on the platinum electrode even after 9 min of Ar^+^ treatment indicative of another decomposition product, which we attribute to S^2−^ (see Figure S7 in the Supporting Information). Previous results of Sharon et al. on the Li−O_2_ system in DMSO‐containing electrolyte showed the presence of higher oxidation states of sulfur on the electrode surface as SO_3_^2−^ and SO_4_^2−^, which was attributed to a side reaction between DMSO and the reactive oxygen species generated during the ORR.^[43]^ In our case, the S 2p core level peak is observed after 9 min of Ar^+^ treatment and therefore arising from a species located close to the Pt electrode. Therefore, the signal should in our case arise from a reaction of the electrocatalyst Pt with DMSO. It is well known that on Pt electrodes adsorption of layers of DMSO as well as further reduction of DMSO occurs.[^44^, ^45^, ^46^] Overall, the decomposition mechanism of DMSO by reactive oxygen species generated during the ORR is still unclear. One of the best suggestions is the decomposition of the electrolyte by the highly reactive, electronically exited state of oxygen, that is, singlet oxygen. Singlet oxygen was found to be a product during the ORR in organic solvents due to the disproportionation of superoxide compounds.^[34]^

**Figure 9:**
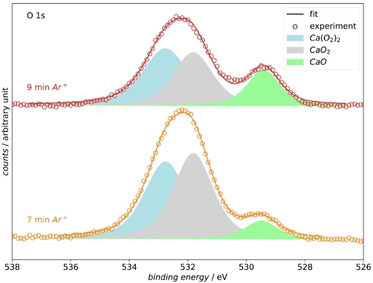
Deconvolution of the O 1s region of the spectra collected from the Pt electrode after 7 min Ar^+^ treatment (yellow) and 9 min Ar^+^ treatment (also shown in Figure 7). The experimental data is shown as circles and the resulting fit is plotted as line. The different deconvoluted species [Ca(O_2_)_2_, CaO_2_, and CaO] are plotted as filled curves under the experimental data. The at % of the different deconvoluted peaks are shown in Table 2.

The O 1s region is deconvoluted for the experiments after 7 and 9 min Ar^+^ treatment (see Figure 9). We chose those two experiments for the analysis, due to the lack of oxygen‐containing carbon compounds as we showed before. Therefore, the deposited layer consists exclusively of calcium−Oxygen compounds

The O 1s region in Figure 9 reveals a new peak at 529.4 eV binding energy after 7 min Ar^+^ treatment, which was deconvoluted into the light green peak, which is assigned to CaO (see also literature overview in Figure 7).^[47]^ Oxides were previously also found on electrodes of lithium–air systems.[^48^, ^49^] The origin of the oxide formation in these systems is still unclear. We assume that the oxide is formed only as adsorbate in the monolayer range, as our previous DEMS measurements do not show evidence for a significant occurrence of the 4 e^−^‐process.[^13^, ^15^] The formation of CaO might also occur as artefact from the XPS analysis. In the Supporting Information we summed up our arguments why we exclude the formation of CaO as an artefact from the XPS analysis procedure.

Regarding the peak towards higher binding energies in the O 1s region, the high FWHM of 2.34 eV in Figure 7 of the peak after 5 min Ar^+^ treatment suggests that several species contribute to this peak. Therefore, we deconvoluted the peak into a CaO_2_ contribution (grey peak in Figure 9) and a Ca(O_2_)_2_ contribution (blue peak in Figure 9). The assignment towards CaO_2_ and Ca(O_2_)_2_ is made by taking the binding energy into account as well as calculating the stoichiometry of the calcium−Oxygen compound from the XPS data. Based on our analysis of the different binding energies of calcium−oxygen compounds there are two possible species that can contribute to the intensity in the binding energy region of the grey peaks in Figure 9: Ca(OH)_2_ and CaO_2_. In our literature search concerning the binding energy of CaO_2_ we only found one value for the binding energy at 531.9 eV.^[50]^ Since the number of transferred electrons per oxygen molecule is slightly higher than 1 e^−^/O_2_ on a Pt electrode during the ORR^[13]^ it is plausible to assume that also minor amounts of peroxide are formed. Moreover, the precipitation of CaO_2_, which is generated from the chemical disproportionation of Ca(O_2_)_2_ (as was shown above in the context of Figure 5), is also a possible origin of CaO_2_ on the surface. The results concerning the formation of hydroxides during the ORR in DMSO in literature are equivocal. The stability of DMSO in a Li−O_2_ cell was studied extensively. There are reports that LiOH can be formed from LiO_2_ and Li_2_O_2_ in presence of DMSO.[^43^, ^51^, ^52^] On the other hand, there are reports that DMSO is a stable electrolyte in a Li–air cells.[^42^, ^53^] The formation of LiOH in these systems is observed on a timescale of 100–500 h. If the reactivity of CaO_2_ and Ca(O_2_)_2_ is comparable to the Li‐containing compounds, we would conclude that the Ca(OH)_2_ is not formed in our experiment (timescale 1 h). Therefore, an assignment of the grey peak in Figure 9 as CaO_2_ is conclusive. Concerning the blue peaks in Figure 9: To our knowledge, there are no binding energies of the O 1s core level of Ca(O_2_)_2_ reported in literature. From a chemical point of view the O 1s binding energy of Ca(O_2_)_2_ should be shifted positive compared to CaO_2_, which is the case in the assignment of Figure 9.

The presence of Ca(O_2_)_2_ on the surface becomes obvious if the stoichiometry of the calcium−Oxygen compound is calculated from the intensity of the Ca 2p and O 1s core‐level excitations. The calculated O/Ca ratios as well as the amount of different oxygen species resulting from the deconvolution are shown in Table 2; for details of the calculation see the Supporting Information.

**Table 2:** Peak areas of the O 1s region of the deconvoluted spectra in Figure 9. The calculated O/Ca is also shown.^[a]^

*t*(Ar^+^) [min]	CaO [at %]	Peroxide [at %]	Superoxide [at %]	O/Ca
7	8.2	46.9	44.9	2.97
9	19.9	35.9	44.2	3.12

[a] For the calcultaion of O/Ca only the areas of the peroxide and superoxide region were used. The area of the Ca 2p peaks was corrected over the expected amount of Ca calculated form the deconvoluted CaO O 1s peak.

Table 2 shows that the O/Ca ratio for 7 and 9 min Ar^+^ treatment is approximately 3. For the compounds of interests, Ca(O_2_)_2_ and CaO_2_, the expected ratio of O/Ca is 4 and 2, respectively. Therefore, a ratio of 3 indicates the presence of Ca(O_2_)_2_ on the surface and moreover a nearly equal distribution of peroxide and superoxide. This calculated O/Ca ratio was also used in the deconvolution routine to define the ratio of deconvoluted peak areas after 7 and 9 min Ar^+^ etching (see Figure 9). It can be seen that by doing this, the deconvolution is represents the experimental data well.

The XP spectrum of the transferred Pt electrode after sweeping the potential into the OER region is shown in Figure 6. The survey spectrum shows that the surface now mainly consists of platinum. The Ca 2p core‐level peaks are not visible anymore. This shows that all deposited calcium species can be stripped from the Pt surface by applying a high electrode potential, that is, 0.7 V vs. Ag^+^/Ag. The remaining contaminants on the surface now are mainly aliphatic carbon at a binding energy of 284.8 eV and oxygen‐containing carbon species at C 1s binding energies >285.5 eV accompanied by a O 1s signal at 531.4 eV (see C 1s and O 1s region in Figure 6). Aliphatic carbon is a well‐known contaminant in XPS experiments. The origin of the oxygen‐containing carbon species is probably the exposure of the electrode to the glovebox atmosphere, which contains organic solvent vapors, and contaminates adsorbed from the organic electrolyte. Therefore, we would conclude that the electrocatalyst Pt was fully regenerated by sweeping the electrode potential into the OER.

### Interpretation of the mechanism of the ORR in Ca^2+^‐containing DMSO

It is rather surprising that according to the XPS results the same Ca−O species is present on Au as well as on Pt since our previous investigations showed fundamental differences with respect to the reduction mechanism on these two electrocatalysts.^[13]^ At gold initially a 2‐electron process occurs, while at platinum a 1‐electron process is observed over the whole potential (and time) range. Moreover, the current transients in Figure S4 also imply that a different number of electrons are transferred and that the reduction mechanism, especially in the beginning of the ORR, is fundamentally different for both electrodes. While there seems to be a blocking effect that alters the reaction mechanism to a 1‐electron process also on Au, the RRDE experiments show that the blocking of the electrode stops after the transition to the 1‐electron process and a diffusion‐limited current is exhibited (see Figure 2). The transition of the mechanism of the ORR in DMSO‐based electrolytes from the 2 e^−^ process to the 1 e^−^ process was already observed in Li^+^‐containing solution.[^28^, ^54^, ^55^] There, this observation was explained with a geometric effect of the deposited peroxide layer: The deposited peroxide covers adsorption sites on the electrocatalyst, which are needed to reduce oxygen to peroxide. However, in contrast to the observations in Li^+^‐containing electrolytes, the electrodes in the presence of Ca^2+^ are not fully blocked (the 1‐electron process is maintained within a sweep), which indicates a significant difference between the deposition mechanisms of Li_2_O_2_ and CaO_2_. Based on the knowledge of the Li^+^‐containing system, we explain the apparent contradiction between the different reaction mechanisms and the observation of the same chemical species on the Au and Pt surfaces.

Au surfaces also showed an exclusive reduction path to reduce oxygen to peroxide in the Li^+^‐containing DMSO.^[28]^ We suspect that there is a similarity concerning a direct reduction of oxygen to peroxide on Au electrocatalysts in Ca^2+^‐containing DMSO, as it was previously observed in Li^+^‐containing DMSO. We would like to postulate this statement here, since of course extensive kinetic measurements are necessary to confirm this statement.

On the Au electrode we observe a transition from the 2‐electron process to the 1‐electron process after a certain time. We believe that this is because adsorption sites, which are needed to perform the 2 e^−^ process, are blocked by a strongly adsorbed layer of either peroxide or, more probable according to the XPS results, of CaO. CaO is likely to form at lower electrode potentials and, if formed as a small percentage of the overall ORR products, it would accumulate on the electrode surface as it is believed to be insoluble in the DMSO‐based electrolyte. This is also supported by the fact that the electrocatalyst could only be reactivated by applying higher potentials and not by allowing slow dissolution of a superoxide or peroxide layer. The electrochemical oxidation of CaO is plausible as the standard potential of the oxidation (3.35 V vs. Ca^2+^/Ca)^[56]^ is reached in the experiment. Nevertheless, the electrode remains active to maintain the 1‐electron process after partial blocking. Therefore, on both electrodes the main product of ORR is calcium superoxide. However, we also found appreciable amounts of calcium peroxide in our XPS measurements. Due to the disproportionation reaction of Ca(O_2_)_2_ it is likely that CaO_2_ particles are formed on the Pt electrode. After an equilibrium time a final state is reached on both electrodes. The same amount of CaO_2_ and Ca(O_2_)_2_ is formed. Underneath the superoxide and peroxide layer a layer of CaO is located. Moreover, the RRDE results in Figure 2 show that the superoxide is readily transported to the ring electrode, suggesting that it is much better soluble than the peroxide. Only in the absence of convection it would also accumulate on the surface. Especially for the superoxide this would also hinder the further disproportionation to peroxide. The stability of solid calcium superoxide was already reported.[^57^, ^58^] This would explain why we observed superoxide in the XPS experiments. On top of the superoxide and peroxide layer a layer of solvent decomposition products is located. This layer could also hinder the dissolution process of the superoxides and peroxides.

### ORR mediation by DBBQ in Ca^2+^‐containing DMSO

Within the Li−O_2_ community, redox mediators for ORR have become common as they prevent the sudden‐death phenomenon due to blocking/passivation of the electrode surface by a solvent‐mediated ORR mechanism.[^21^, ^59^, ^60^, ^61^] Despite the solubility of calcium superoxide and calcium peroxide, it can be anticipated that the use of redox mediators is advantageous for the ORR in Ca^2+^ electrolytes for the following reasons:

1. If a redox mediator is used, the ORR potential can be shifted positively.[^21^, ^60^, ^61^]
2. It has been observed that the use of redox mediators reduces the amount of undesired side reactions.^[62]^

**Figure 10:**
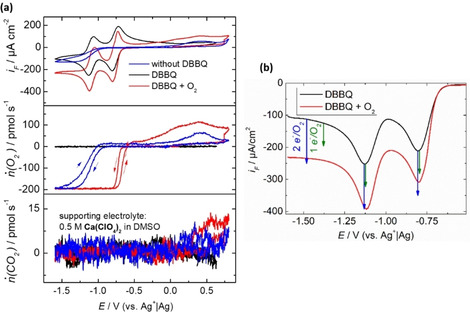
a) CVs, O_2_ flux, and CO_2_ flux in a 0.5 m Ca(ClO_4_)_2_ solution in DMSO. The blue‐traced measurements were recorded in the absence of DBBQ. In the black‐traced measurements (deoxygenated solution) and in the red‐traced measurements (solution saturated with 700 mbar O_2_) 7.5 mm DBBQ was added to the supporting electrolyte. The applied sweep rate was 10 mV s^−1^. We used a porous Teflon membrane sputtered with Au as working electrode. b) Magnification of the cathodic sweep of the CVs in a DBBQ containing solution shown in (a). The arrows indicate the increase in the reduction current based on 1 e^−^/O_2_ (green) and 2 e^−^/O_2_ (blue) reduction. This calculation was made with the observed diffusion limited oxygen consumption of 200 pmol s^−1^_._

Therefore, the ORR in Ca^2+^‐containing DMSO mediated by DBBQ was also investigated using DEMS and XPS. The DEMS measurements are presented in Figure 10.

In a deoxygenated solution, the CV of DBBQ shows two reversible peak pairs a (see black‐traced measurement in Figure 10a). We would like to point out that it is due to the special thin‐layer construction of the DEMS cell that in the CV diffusion‐limited currents are observed for the oxygen reduction in the absence of convection (for more details see Ref. [21]). In the presence of oxygen, the decrease of the oxygen flux into the vacuum of the MS at the onset potential of the DBBQ reduction shows that the DBBQ monoanion mediates the ORR in a 0.5 m Ca(ClO_4_)_2_ in DMSO. The CV of DBBQ in the presence of oxygen is shifted towards lower currents. This shift can be explained in analogy to the postulated mediation mechanism in Li^+^‐containing electrolytes.^[60]^ The mechanism is formulated for Ca^2+^‐containing electrolytes is presented in Equations (1)–(3) (here for the DBBQ monoanion):

(1) $$DBBQ_{\left(sol\right)}+e^{-}\overset{\;\;\;\;}{\underset{}{\to }}\;DBBQ_{\left(sol\right)}^{-}$$

(2) $${Ca}_{\left(sol\right)}^{2+}+DBBQ_{\left(sol\right)}^{-}\overset{\;\;\;\;}{\underset{}{\to }}\;{\left\{Ca\cdot \cdot \cdot DBBQ_{\left(sol\right)}\right\}}^{+}$$

(3) $${\left\{Ca\cdot \cdot \cdot \;DBBQ_{\left(sol\right)}\right\}}^{+}+O_{2\left(sol\right)}\overset{\;\;\;\;}{\underset{}{\to }}\;{\left\{Ca\cdot \cdot \cdot DBBQ_{\left(sol\right)}\cdot \cdot \cdot O_{2}\right\}}^{+}$$

DBBQ is reduced at the electrode [reaction (1)]. The reduced DBBQ species forms an ion pair with Ca^2+^ [reaction (2)]. This ion pair formation is well known for benzoquinones[^63^, ^64^] and was also investigated by us in the context of the ORR in Li^+^‐containing electrolyte.^[61]^ It is expected that the DBBQ ion pair forms a mediator−Oxygen complex [reaction (3)]. This is followed by the formation of peroxides or superoxides by the following Equations (4)–6:

(4) $$2\;{\left\{Ca\cdot \cdot \cdot DBBQ_{\left(sol\right)}\cdot \cdot \cdot O_{2}\right\}}^{+}\overset{\begin{array}{c} \;\;\;\;\\ \;\; \end{array}}{\underset{}{\to }}CaO_{2\left(s\right)}+O_{2\left(sol\right)}+2\;DBBQ_{\left(sol\right)}+Ca_{\left(sol\right)}^{2+}$$

(5) $$\;{\left\{Ca\cdot \cdot \cdot DBBQ_{\left(sol\right)}\cdot \cdot \cdot O_{2}\right\}}^{+}+{\left\{Ca\cdot \cdot \cdot DBBQ_{\left(sol\right)}\right\}}^{+}\overset{\;\;\;\;}{\underset{}{\to }}CaO_{2\left(s\right)}+2\;DBBQ_{\left(sol\right)}+\;Ca_{\left(sol\right)}^{2+}$$

(6) $$\;{\left\{Ca\cdot \cdot \cdot DBBQ_{\left(sol\right)}\cdot \cdot \cdot O_{2}\right\}}^{+}\overset{\;\;\;\;}{\underset{}{\to }}CaO_{2\left(sol\right)}^{+}+DBBQ_{\left(sol\right)}$$

In reactions (4)–(6) the mediator, DBBQ, is regenerated. The regenerated DBBQ can diffuse to the electrode and be reduced again, which explains the decrease in the current in the presence of O_2_. Taking the consumption of oxygen and the electrons flowing into the reduction of DBBQ into account, one can calculate the expected ratio between transferred electrons per oxygen molecule on the basis of the postulated reactions. This results in 2 e^−^/O_2_ for reactions (4) and (5) and 1 e^−^/O_2_ for reaction (6). For reaction (6), a following disproportionation reaction of the superoxide is plausible as we also showed in the beginning of this paper (see Figure 5). Based on the experimentally observed oxygen consumption in the diffusion‐limited region (oxygen flux of 200 pmol s^−1^), the expected increase of the faradaic current was calculated for 1 e^−^/O_2_ and 2 e^− ^/O_2_ and is shown as arrows in Figure 10b. The comparison to the experimental reduction waves of DBBQ in presence and absence of oxygen shows that decrease of the reduction current in presence of oxygen results in a change in the mediated ORR mechanism. In the first reduction peak of DBBQ we observe a mixed process between the 1 e^−^/O_2_ and 2 e^−^/O_2_ process. In the second reduction peak and in the diffusion‐limited region mainly the 2 e^−^/O_2_ process is observed. Overall, the mediated ORR is shifted 360 mV towards more positive electrode potentials (compare blue and red measurements in Figure 10a).

At higher electrode potentials the comparison of the CVs and the MS data shows that the positive current is due to the oxidation of oxygen‐releasing species such as Ca(O_2_)_2_ and CaO_2_. The oxidation of CO_2_‐releasing species is also observed in the DBBQ‐containing measurement at electrode potentials higher than 0.5 V vs. Ag^+^/Ag.

In the DBBQ‐containing electrolyte the surface of an Au electrode was analyzed by XPS in the same manner as in the DBBQ‐free solution: The potential was held for 60 min at −1.5 V vs. Ag^+^/Ag, and afterwards the electrode was washed with DMSO and transferred to the XPS. The corresponding XP spectra are shown in Figure S9 in the Supporting Information. The XPS analysis shows that after the transfer an overlayer, which mainly consists of ORR decomposition products (C−O, O−C=O, and CO_3_^2−^), was deposited on the Au surface. This overlayer is rather thin since it can be removed after 120 s of Ar^+^ etching. Most of the Au surface is recovered after the first Ar^+^ etching stage, which is different from the experiments without the mediator. This indicates that the deposited layer in the DBBQ‐containing electrolyte is thinner than in the DBBQ‐free solutions. After the first Ar^+^ treatment of the surface, we still observe Ca_*x*_O_*y*_ species with an overall high intensity of the Au 4f XPS peaks. This suggests that the main part of the surface is free Au and that particles are deposited on the surface. The formation of large particles in the mediated ORR is well known.[^60^, ^65^] A comparison to the XP spectra in DBBQ‐free solution shown above reveals that these particles have the same chemical composition, that is, CaO_2_, Ca(O_2_)_2_, and CaO.

We also investigated the DBBQ‐mediated ORR in Mg^2+^‐containing solution (see DEMS measurements in Figure S10 in the Supporting Information). There, we also observe a beneficial ORR potential shift of 280 mV. Unfortunately, DBBQ is not a reversible redox system in the presence of Mg^2+^ and therefore not suited as mediator for the ORR.

## Conclusions

In this study we report several findings concerning the oxygen reduction reaction (ORR) in Ca^2+^‐containing DMSO. The apparent collection efficiency as determined by rotating ring disk electrode (RRDE) shows that the mechanism of the ORR on Au changes from a mixed process of O_2_^2−^ and O_2_^−^ formation to an exclusive O_2_^−^ formation. The unusually high charges for a metal‐air system in a non‐aqueous solvent observed during the ORR in the RRDE experiments and in the potential‐step experiment suggest that the main products of the ORR are soluble in DMSO and therefore do not poison the electrode surface. The solubility of CaO_2_ together with the possibility to reoxidize it was proven using a thin‐layer differential electrochemical mass spectrometry (DEMS) cell. Poisoning only occurs very slowly due to a layer of ORR products strongly attached to the electrocatalyst (CaO or strongly bound superoxide/peroxide species), which is only removed at higher electrode potentials (0.3 V vs. Ag^+^/Ag), thus regenerating the electrocatalyst. The disproportionation reaction of O_2_^−^ in the presence of Ca^2+^ was demonstrated via MS. This is accompanied by the evolution of several side products. We assume that these side products are generated by a reaction with ^1^O_2_, which was reported to be a product besides ^3^O_2_ during the disproportionation reaction in the presence of Li^+^. The formation of side products during the disproportionation is, on the one hand, a problem for metal‐air technologies in general. On the other hand, the formation of CaO_2_ from the disproportionation would boost the theoretical gravimetric energy density form 838 Wh kg^−1^ (superoxide as discharge product) to 2515 Wh kg^−1^. With XPS the surface chemistry of a thin film, which was deposited on Pt and Au electrodes, was investigated. The following conclusion is made: The top layer of the film contains decomposition products such as CO_3_^2−^ and other oxygen containing carbonaceous species. During Ar^+^ etching Ca(O_2_)_2_, CaO_2_, and CaO were found on the surface. On Au and Pt electrodes the same species are deposited. Sweeping the potential into the OER and performing an ex situ XPS measurement shows that the surface of a Pt electrode is fully regenerated. Furthermore, the functionality of 2,5‐di‐*tert*‐1,4‐benzoquinone (DBBQ) as redox mediator for the ORR in Ca^2+^‐containing DMSO was investigated. The ORR in the presence of DBBQ benefits from a 36 mV positive potential shift compared to the bare electrolyte. An analysis of the number of transferred electrons per oxygen molecule shows a transition from a mixed process of O_2_^2−^ and O_2_^−^ formation (during the generation of DBBQ^−^) to an O_2_^2−^ formation (during the generation of DBBQ^2−^). Ex situ XPS measurements of the electrode surface after the ORR in the DBBQ‐containing electrolyte measurements show that a thinner film (compared to the bare electrolyte) was deposited. The same species were found on the electrode surface as in the absence of DBBQ.

## Conflict of interest

The authors declare no conflict of interest.

## Supporting information

As a service to our authors and readers, this journal provides supporting information supplied by the authors. Such materials are peer reviewed and may be re‐organized for online delivery, but are not copy‐edited or typeset. Technical support issues arising from supporting information (other than missing files) should be addressed to the authors.

Supplementary
