# Supplementary material for: The Oxygen Reduction Reaction in Ca2+‐Containing DMSO: Reaction Mechanism, Electrode Surface Characterization, and Redox Mediation
Source: ChemSusChem. 2020 Sep 18;14(1):428–40. doi: 10.1002/cssc.202001605 (PMC7821240; doi:10.1002/cssc.202001605)
Supplement: Supplementary file 1 — Supplementary [file CSSC-14-428-s001.pdf]

ChemSusChem

Supporting Information

The Oxygen Reduction Reaction in Ca^{2+}-Containing DMSO: Reaction Mechanism, Electrode Surface Characterization, and Redox Mediation**

Pawel Peter Bawol, Philip Heinrich Reinsberg,* Andreas Koellisch-Mirbach, Christoph Johannes Bondue, and Helmut Baltruschat© 2020 The Authors. Published by Wiley-VCH GmbH. This is an open access article under the terms of the Creative Commons Attribution License, which permits use, distribution and reproduction in any medium, provided the original work is properly cited.

Table of Contents

1. Mass spectrometric measurement of the superoxide disproportionation 2

1.1. Sketch of the experimental setup 2

1.2. Full mass scans during the disproportionation reaction 2

1.3. Carbonate formation during the disproportionation reaction 4

2. Preparation of the XPS electrodes 5

3. Survey XPS Spectra 7

4. Deconvolution of the C1s region after the ORR on a Pt electrode 9

5. Literature Research towards binding energies of Calcium Oxygen Compounds 10

5.1.Calciumcarbonat 10

5.2. Calciumhydroxide 10

5.3. Calciumoxide 11

6. XPS investigation of the products of the ORR mediated by DBBQ 12

7. Procedure to determine the stoichiometry of the Ca-O compound with XPS 13

9. The effect of Mg^{2+} on the reduction of DBBQ in DMSO based electrolyte 15

10. Photograph of the used DEMS electrode 16

1. **Mass spectrometric measurement of the superoxide disproportionation**

1.1. Sketch of the experimental setup

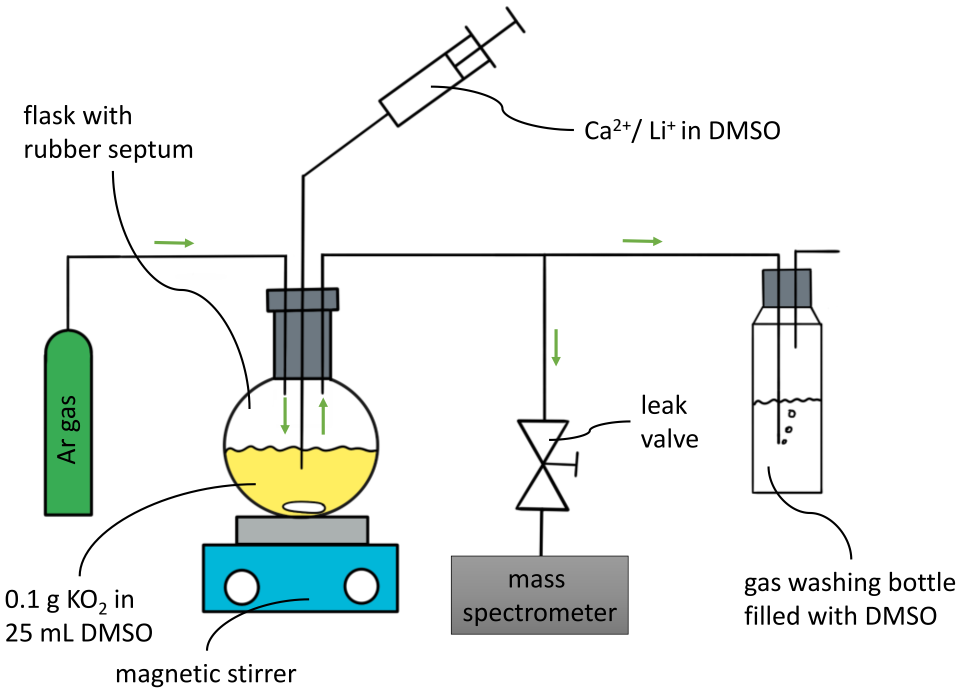

Figure S 1: Schematic sketch of the experimental set up, which was used to detect the gaseous products via mass spectrometry during the disproportionation of superoxide in DMSO.

1.2. Full mass scans during the disproportionation reaction

In order to find out whether other gases are also produced during the disproportionation, mass scans of m/z=1-100 were taken in chronological order. Figure S 2 shows the mass scans in a 2D plot. For a better presentation of the measurement results, the signals caused by the Ar carrier gas (m/z=20.40) were removed from the data series. To guarantee a better visibility of the signals, an intensity normalization was carried out: All signals were normalized to the most intense signal for m/z=44. Then the signal of m/z=32 was internally normalized to its maximum again.

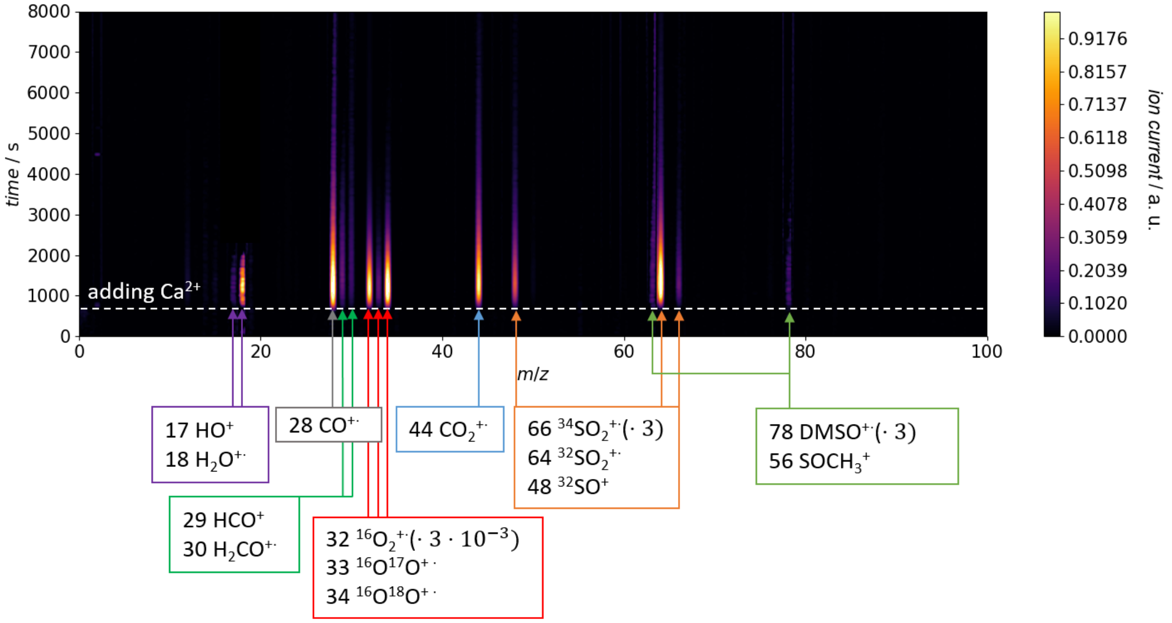

Figure S 2: Mass scans from m/z=1 to 100 versus time. The dashed white line corresponds to the time at which 3 mL of 1 M $Ca(ClO_4)_2$ solution in DMSO were added to 0.1 g KO_2 in 25 mL DMSO. Ar was used as carrier gas and the experimental setup is sketched in Figure S 1. The intensity is given in arbitrary units and the procedure of intensity calculation is described in the text.

As can be seen in the Figure S 2, a number of other signals are observed in addition to the oxygen signal (m/z=32). A possible assignment to the different chemical species can also be found in Figure S 2. The formulation of a mechanism how these compounds are formed during disproportionation is currently not possible. The most plausible source would be a side reaction with singlet oxygen, which was observed as a by-product of disproportionation in significant amounts [1] and is a highly reactive species.

An origin of the signal of mass 28 could also be an introduced contamination of N_2 by adding the electrolyte. We would argue that we should then observe a comparable signal for mass 28 after adding the Li^+ containing solution, which is not the case (see Figure 5 in the main manuscript).

The increase of the signal on mass 18 is probably related to the higher water content of the DMSO solution containing $Ca(ClO_4)_2$ as compared to the DMSO solution containing KO_2. Due to the

bubble formation during disproportionation, the rate of water evaporation into the mass spectrometer is also increasing

1.3. Carbonate formation during the disproportionation reaction

It was already reported, that another side product during the disproportionation are carbonates [1, 2]. It is most likely that that generated singlet oxygen during the disproportionation reaction is undergoing side reactions with the solvent to produce carbonates. *Figure S 3* shows that an acidification of the solution after oxygen has been produced by the disproportionation of superoxide triggered by Ca^{2+} leads to CO_2 formation. The protonation of carbonates is believed to be the source of the CO_2 evolution [3]. Therefore we would also conclude the presence of carbonates after the disproportionation of superoxide in the presence of Ca^{2+}. We also observe a parallel O_2 evolution to the CO_2 evolution. A possible reason could be that the generated carbonates are deposited on the KO_2 surface and are therefore hindering a further disproportionation reaction. By adding H_2SO_4 to the solution the carbonates are consumed and the disproportionation can start again.

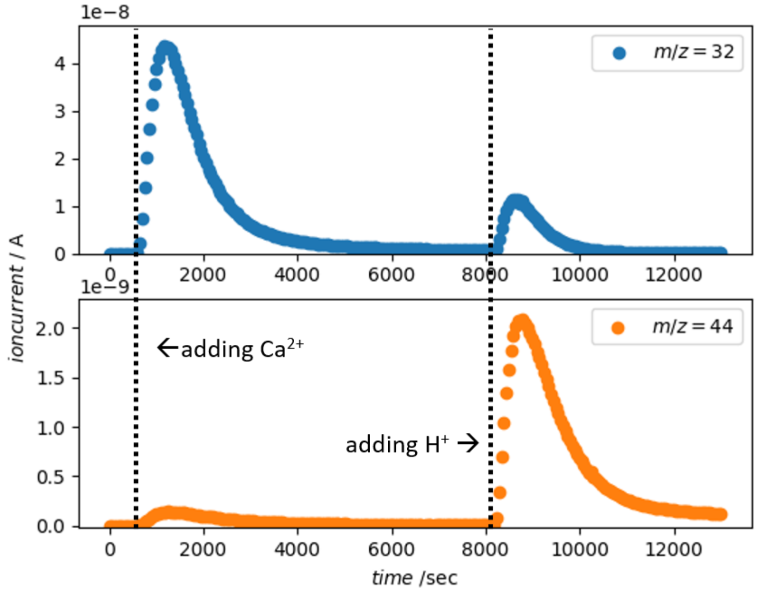

Figure S 3: Ionic current of O_2 (blue, *m/z*=32) and CO_2 (orange, *m/z*=44) as a function of time. The gas phase over a stirred solution containing 0.1 g KO2 in 25 mL DMSO was analyzed by MS after adding 3 mL of 1 M $Ca(ClO_4)_2$ in DMSO and 0.5 M H_2SO_4 in H_2O. The time at which the Ca^{2+} and H^+ containing solutions were added, are indicated as dashed line in the graphs.

2. Preparation of the XPS electrodes

To investigate the precipitation of solid products on Pt and Au electrodes during the ORR in Ca^{2+} containing DMSO XPS studies were carried out. As we showed in a previous study [4] and in the main paper(see Figure 2 and Figure 3 in the paper), the ORR in Ca^{2+} containing DMSO is mainly resulting in soluble products. Therefore, to accumulate species on Au and Pt surfaces the ORR was performed by holding the potential at -1.5 V vs. $Ag^+|Ag$ for 60 mins in 0.2 M $Ca(ClO_4)_2$ in DMSO. The resulting current transients are displayed in Figure S 4.

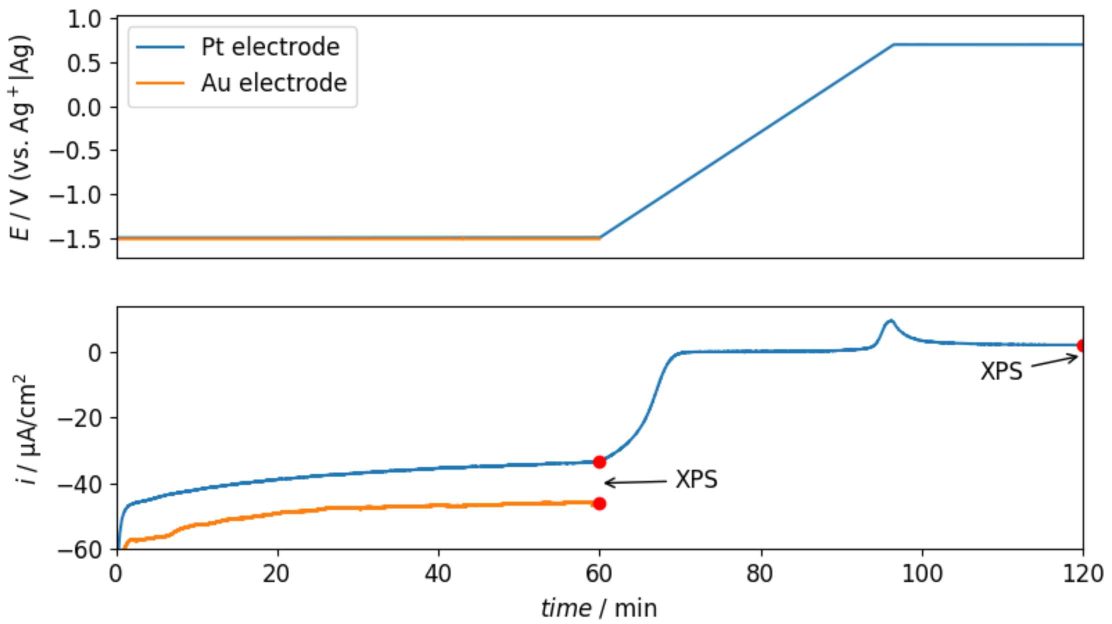

Figure S 4 Potential versus time (top figure) and resulting current transient (bottom) in a 0.2 M $Ca(ClO_4)_2$ electrolyte in DMSO. As working electrode, Pt and Au were used (figure caption). All experiments were performed in a glovebox. The electrolyte was saturated with a 20% O_2 mixture

in Ar. The red circles are indicating at which time the samples were transferred to the XP analysis chamber.

After an initial decrease of the current due to double-layer charging, the current reaches a slightly decreasing plateau within the first 100 s. This plateau, which most probably refers to a two-electron transfer, is followed by an abrupt decrease in current probably due to a transition of the two- to a one-electron process (compare with the RRDE results in the beginning of this paper). It is interesting to note that the current during the first plateau does not follow the behavior predicted by the Cottrell-equation, which implies that the reaction is not simply limited by mass-transfer in the bulk but maybe by the rate of precipitation in analogy to the findings in the Li^+-containing system. After that, the current decreases only slightly over time indicating a diffusion-limited process. In the time scale of the experiment no complete blocking of the electrode surface was observed, as we would expect in a similar experiment in a Li^+ containing system [5]. This again shows that in the Ca^{2+} system mainly soluble species are generated or that the species are not blocking the electrode/preventing the reaction from happening. After 60 min holding the potential in the ORR region the electrodes were transferred to the UHV chamber to record the XP spectra. With the Pt electrode an additional experiment was performed: After holding the potential at -1.5 V vs $Ag^+|Ag$ the potential was swept with 1 mV/s to 0.7 V vs $Ag^+|Ag$, where the potential was held again. At positive electrode potentials the current transient is showing an oxidation peak. This suggests that species, which are deposited on the electrode surface during the ORR, are oxidized. The overall minor oxidative charge compared to the reductive charge is again implying that the main products of the ORR are soluble and are not deposited on the electrode surface. The oxidation of soluble ORR products is in our case not likely to be visible due to the large electrolyte excess of

35 mL in the electrochemical cell. After a total time of 120 min the experiment was stopped and the electrode was again transferred into the XP spectrometer.

3. Survey XPS Spectra

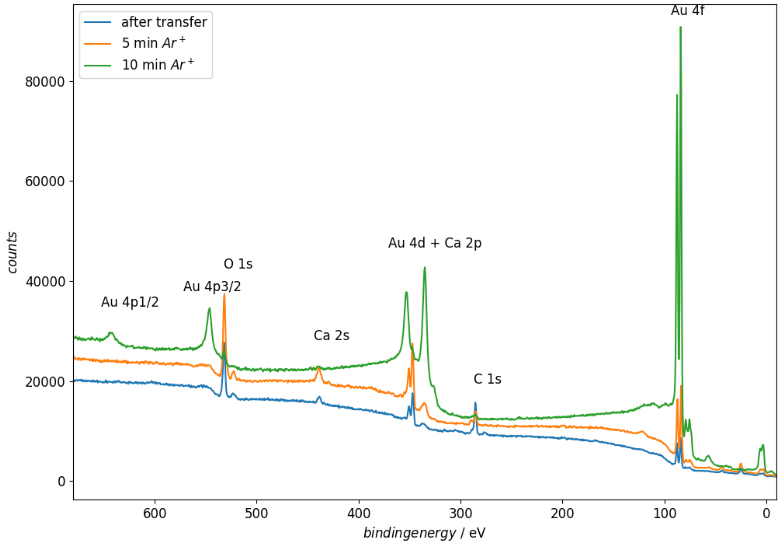

Figure S 5: Survey XP Spectra of a Au electrode after performing the ORR in 0.2 M $Ca(ClO_4)_2$ in DMSO for 60 min.

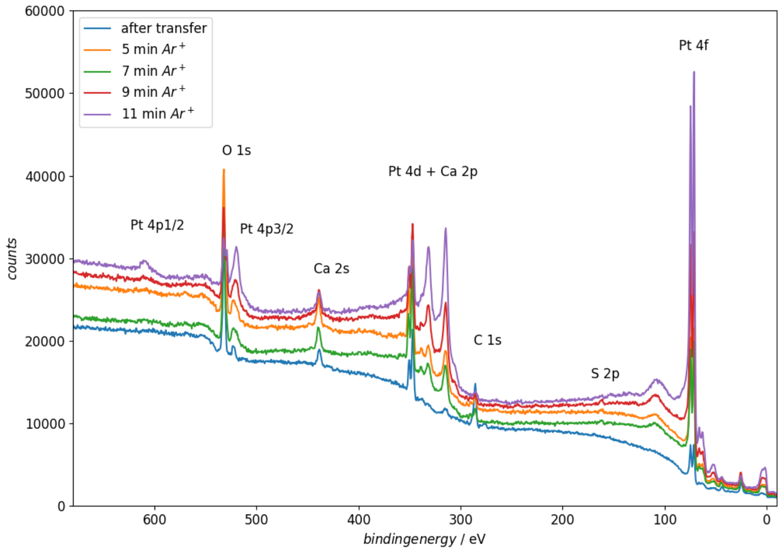

Figure S 6. Survey XP Spectra of a Pt electrode after performing the ORR in 0.2 M $Ca(ClO_4)_2$ in DMSO for 60 min.

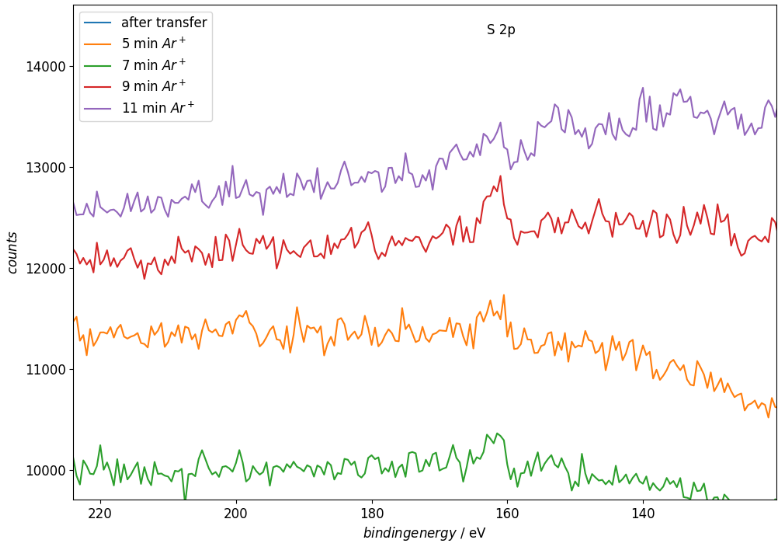

Figure S 7: Magnification of the S2p region out of Figure S 6.

4. Deconvolution of the C1s region after the ORR on a Pt electrode

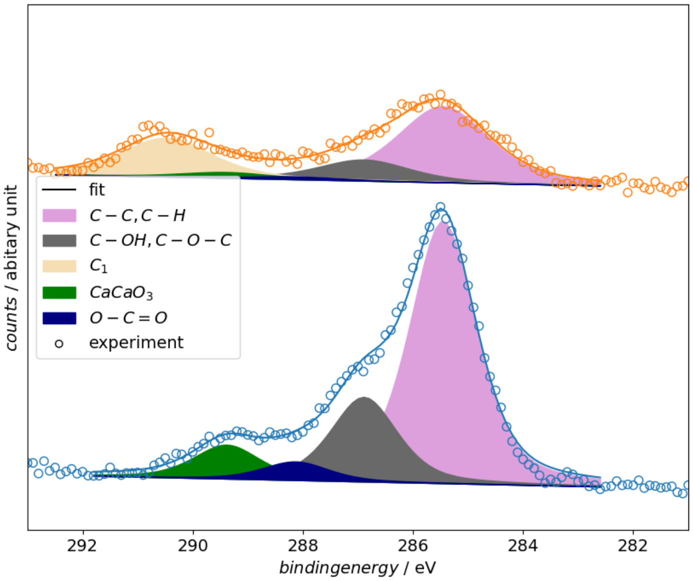

Figure S 8 Deconvolution of the C 1s region of the spectra collected from the Pt electrode after transfer (blue) and 5 min Ar^+ treatment). The experimental data is shown as circles and the resulting fit is plotted as line. The different deconvoluted species are plotted as filled curves under the experimental data.

Figure S 8 is showing the deconvolution of the C 1s region into different chemical compounds (see labels and the discussion in the paper). We also observed a peak at 291 eV binding energy (see deconvoluted peak C1 in *Figure S 8*). A clear assignment of a species to this peak cannot be made, due to the high binding energy that is usually expected for fluorinated carbon compounds. In our case, no F 1s core level excitation was observed therefore it ca C–F compound is unplausible.

5. Literature Research towards binding energies of Calcium Oxygen Compounds

5.1.Calciumcarbonat

BE(Ca 2p3/2) / eV	*BE(O 1s)* / eV	*BE(C 1s)* / eV	Reference
347	531.4	289.6	[6]
346.8	531.2	289.4	[7]
	531.3		[8]
347.4	531.7	289.8	[9]
346.8	531.2	289.2	[10]
347			[11]
346.7			[12]
347.7		290.1	[13]
347.3			[14]
		289.7	[15]

5.2. Calciumhydroxide

BE(Ca 2p3/2) / eV	*BE(O 1s)* / eV	reference
346.9	531.4	[16]
	533.2	[17]
	532.2	[18]
	530.8	[19]

5.3. Calciumoxide

BE(Ca 2p3/2) / eV	*BE(O 1s)* / eV	reference
	531.3	[8]
346.1	529.4	[20]
347.3	530.1	[21]
346.1	529.0	[9]
347	531.5	[22]
346.8	531.4	[10]
346.65		[23]
346.1		[24]

6. XPS investigation of the products of the ORR mediated by DBBQ

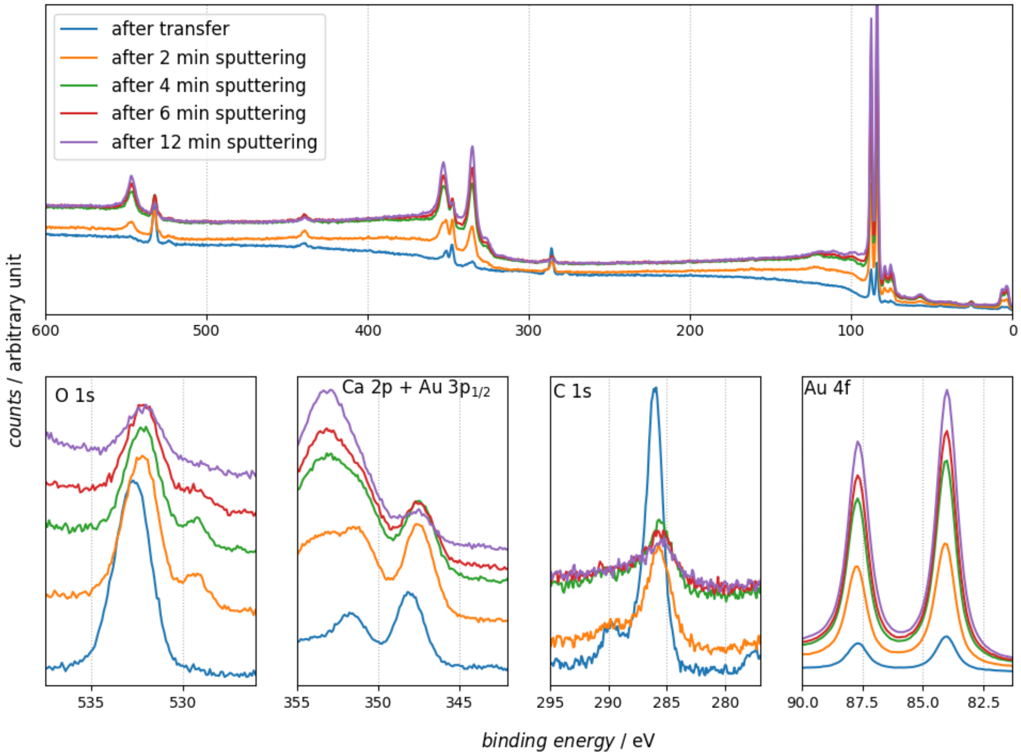

Figure S 9: Survey and high resolution XP spectra of an AU electrode after performing the ORR in a 0.2 M $Ca(ClO_4)_2$ in DMSO with 5 mM DBBQ. Ar+ etching accompanies the XP measurements. For the Ar^+ etching times see the figure caption.

7. Procedure to determine the stoichiometry of the Ca-O compound with XPS

To determine the stoichiometry of the oxygen-compound the contribution of CaO has to be subtracted first from the total Ca (2p) intensity (see Figure 7 in the paper). The evaluation is performed as follows. First the area of the peak corrected for the atomic sensitivity factor (ASF) and the CaO contribution $W(Ca\ 2p_{corr})$ is calculated. The amount of CaO can be determined by the respective deconvoluted peak area $A(O\ 1s_{CaO})$ in Figure 9 in the main manuscript. The following equation results for $W(Ca\ 2p_{corr})$.

$$W(Ca\ 2p_{corr}) = \frac{A(Ca\ 2p)}{ASF_{Ca\ 2p}} - \frac{A(O\ 1s_{CaO})}{ASF_{O\ 1s}} \quad (1)$$

In equation (1) $A(Ca\ 2p)$ is the overall integral of the Ca 2p core level excitation and $ASF_{Ca\ 2p}$ and $ASF_{O\ 1s}$ are the atomic sensitivity factors for the Ca 2p and O 1s core level excitation respectively. To subsequently get insights into the stoichiometry of the calcium-oxygen compound one can now calculate the area ratio of the remaining O 1s contribution (without the contribution of the O1s core level excitation from CaO):

$$W(O\ 1s) = \frac{A\left(O\ 1s_{Ca(O_2)_2}\right) + A\left(O\ 1s_{CaO_2}\right)}{ASF_{O\ 1s}} \quad (2)$$

$A\left(O\ 1s_{Ca(O_2)_2}\right)$ and $A\left(O\ 1s_{CaO_2}\right)$ are the integrals of the respective deconvoluted $Ca(O_2)_2$ and CaO_2 peaks in Figure 9 in the main manuscript. It may be confusing that the areas of the deconvoluted peaks are already assigned to the different species in equation (2), since the calculation described here should make the assignment possible in the first place. Therefore, we would like to point out that we did not make the assignment until after the evaluation described here was performed. By calculating the ratio $\frac{W(O\ 1s)}{W(Ca\ 2p_{corr})}$ the stoichiometry of the Calcium oxygen compound was determined. The results are shown in the main manuscript.

8. Excluding CaO as an XPS analysis artefact

In general, it is conceivable that CaO can form as an artefact of radiation damage from carbonates or peroxides. We can rule this out for the following reasons:

- The CaO peak can be recognized as a shoulder in the measurements immediately after the transfer (see Figure 7 in the paper). At this stage in the experiment a radiation damage is not reasonable.
- Carbonates were detected on the electrode surface after the transfer. The following reaction is assumed for the formation of oxides from carbonates: $CaCO_3 \rightarrow CaO + CO_2$. A quantification of the amount of Carbonate on the electrode surface shows that there is an insufficient small amount of CO_3^{2-} to explain the amount of CaO on the surface.
- For the formation of CaO from CaO2 the following reaction is assumed: $2CaO_2 \rightarrow CaO + O_2$. This reaction can be excluded for the following reasons:
    - The formation of CaO from CaO_2 by sputtering or excitation with X-rays was not observed before [25].
    - The reported temperature of 380°C which is needed for the formation of CaO from CaO_2[26], is probably not reached in the experiment. However, it is probable that this reaction is favored at lower temperatures in the UHV. But theoretical calculations show that CaO_2 is a stable compound at lower pressures [27].

9. The effect of Mg^{2+} on the reduction of DBBQ in DMSO based electrolyte

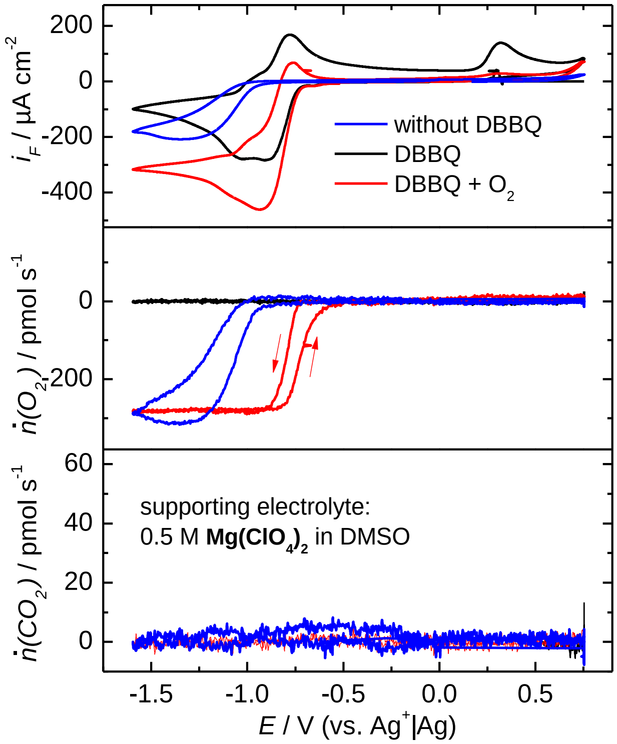

Figure S 10: CVs, O_2 flux and CO_2 flux in a 0.5 M $Mg(ClO_4)_2$ solution in DMSO. The blue traced measurements were recorded in the absence of DBBQ. In the black traced measurements (deoxygenated solution) and in the red traced measurements (solution saturated with 700 mbar O_2) 7.5 mM DBBQ was added to the supporting electrolyte. The applied sweep rate was 10 mVs^{-1}. We used a porous Teflon membrane sputtered with Au as working electrode.

10. Photograph of the used DEMS electrode

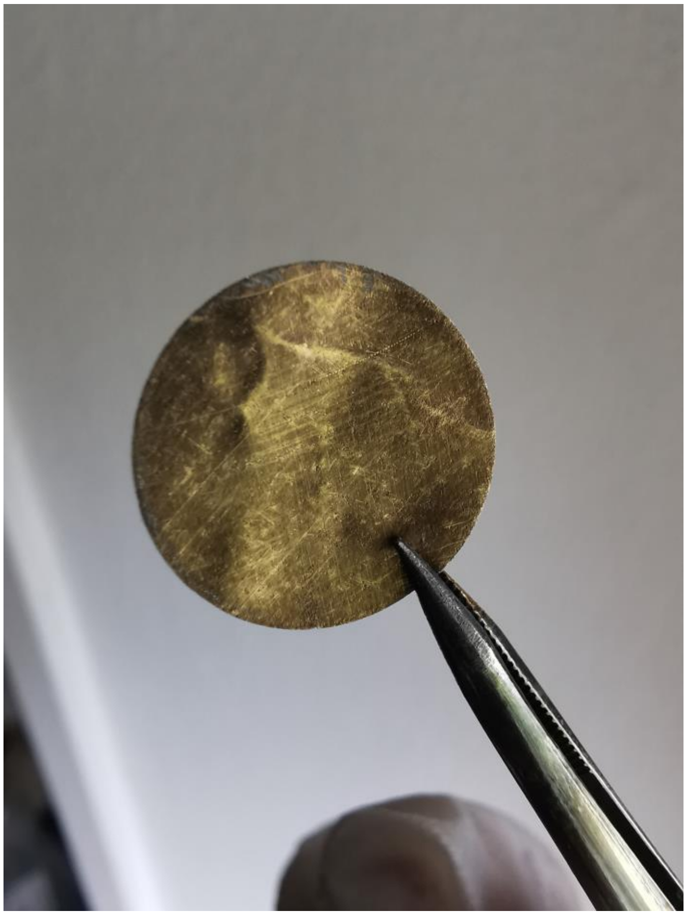

Figure S 11: Photograph of the porous PTFE membrane on which the electrocatalyst (Au, 50 nm) was evaporated.

[1] E. Mourad, Y. K. Petit, R. Spezia, A. Samojlov, F. F. Summa, C. Prehal, C. Leypold, N. Mahne, C. Slugovc, O. Fontaine, S. Brutti, A. Freunberger Stefan, *Energy Environ. Sci.* **2019**, *12*(8), 2559-2568.

[2] L. Schafzahl, N. Mahne, B. Schafzahl, M. Wilkening, C. Slugovc, M. Borisov Sergey, A. Freunberger Stefan, *Angew. Chem.* **2017**, *129*(49), 15934-15938.

[3] N. Mahne, B. Schafzahl, C. Leypold, M. Leypold, S. Grumm, A. Leitgeb, G. A. Strohmeier, M. Wilkening, O. Fontaine, D. Kramer, C. Slugovc, S. M. Borisov, S. A. Freunberger, *Nat. Energy* **2017**, *2*.

[4] P. Reinsberg, C. J. Bondue, H. Baltruschat, *J. Phys. Chem. C* **2016**, *120*(39), 22179-22185.

[5] C. J. Bondue, P. Reinsberg, H. Baltruschat, *Electrochim. Acta* **2017**, *245*(Supplement C), 1035-1047.

[6] A. B. Christie, J. Lee, I. Sutherland, J. M. Walls, *Applications of surface science* **1983**, *15*(1-4), 224-237.

[7] W. J. Landis, J. R. Martin, *Journal of Vacuum Science & Technology A: Vacuum, Surfaces, and Films* **1984**, *2*(2), 1108-1111.
[8] C. D. Wagner, D. A. Zatko, R. H. Raymond, *Anal. Chem.* **1980**, *52*(9), 1445-1451.
[9] M. I. Sosulnikov, Y. A. Teterin, *Doklady Akademii Nauk SSSR* **1991**, *317*(2), 418-421.
[10] B. Demri, D. Muster, *Journal of materials processing technology* **1995**, *55*(3-4), 311-314.
[11] J. F. Moulder, *Physical electronics* **1995**, 230-232.
[12] D. Briggs, *Handbook of X-ray and ultraviolet photoelectron spectroscopy*, Heyden London, **1977**.
[13] S. L. Stipp, M. F. Hochella Jr, *Geochim. Cosmochim. Acta* **1991**, *55*(6), 1723-1736.
[14] Q. Liu, J. S. Laskowski, Y. Li, D. Wang, *Int. J. Miner. Process.* **1994**, *42*(3-4), 251-266.
[15] E. Z. Kurmaev, V. V. Fedorenko, V. R. Galakhov, S. Bartkowski, S. Uhlenbrock, M. Neumann, P. R. Slater, C. Greaves, Y. Miyazaki, *J. Supercond.* **1996**, *9*(1), 97-100.
[16] T. Sugama, L. E. Kukacka, N. Carciello, N. J. Hocker, *Cem. Concr. Res.* **1989**, *19*(6), 857-867.
[17] G. Chiarello, A. Lumachi, F. Parmigiani, P. Ghetti, G. De Michele, *J. Electron Spectrosc. Relat. Phenom.* **1990**, *50*(2), 229-237.
[18] B.-S. Lee, Y.-C. Lin, S.-F. Chen, S.-Y. Chen, C.-C. Chang, *Clinical oral investigations* **2014**, *18*(2), 489-498.
[19] J.-C. Dupin, D. Gonbeau, P. Vinatier, A. Levasseur, *Phys. Chem. Chem. Phys.* **2000**, *2*(6), 1319-1324.
[20] Y. Inoue, I. Yasumori, *Bull. Chem. Soc. Jpn.* **1981**, *54*(5), 1505-1510.
[21] H. v. Doveren, J. A. T. Verhoeven, *J. Electron Spectrosc. Relat. Phenom.* **1980**, *21*(3), 265-273.
[22] T. Hanawa, M. Ota, *Biomaterials* **1991**, *12*(8), 767-774.
[23] H. F. Franzen, J. Merrick, M. Umana, A. S. Khan, D. T. Peterson, J. R. McCreary, R. J. Thorn, *J. Electron Spectrosc. Relat. Phenom.* **1977**, *11*(4), 439-443.
[24] H. Seyama, M. Soma, *Journal of the Chemical Society, Faraday Transactions 1: Physical Chemistry in Condensed Phases* **1984**, *80*(1), 237-248.
[25] K. Zhou, B. Wu, L. Su, X. Gao, X. Chai, X. Dai, *Chem. Eng. J. (Lausanne)* **2017**, *328*, 35-43.
[26] W. Hesse, M. Jansen, W. Schnick, *Prog. Solid State Chem.* **1989**, *19*(1), 47-110.
[27] J. R. Nelson, R. J. Needs, C. J. Pickard, *Phys. Chem. Chem. Phys.* **2015**, *17*(10), 6889-6895.
